# Combining ddPCR and environmental DNA to improve detection capabilities of a critically endangered freshwater invertebrate

**DOI:** 10.1038/s41598-019-50571-9

**Published:** 2019-10-01

**Authors:** Quentin Mauvisseau, John Davy-Bowker, Mark Bulling, Rein Brys, Sabrina Neyrinck, Christopher Troth, Michael Sweet

**Affiliations:** 1 Aquatic Research Facility, Environmental Sustainability Research Centre, University of Derby, Derby, DE22 1GB United Kingdom; 2 Surescreen Scientifics Ltd, Morley Retreat, Church Lane, Morley, DE7 6DE United Kingdom; 3 Freshwater Biological Association, River Laboratory, East Stoke, Wareham, Dorset, BH20 6BB United Kingdom; 4 Natural History Museum, Cromwell Road, London, SW7 5BD United Kingdom; 5 Research Institute for Nature and Forest, Gaverstraat 4, 9500 Geraardsbergen, Belgium

**Keywords:** Freshwater ecology, Conservation biology, Molecular ecology

## Abstract

*Isogenus nubecula* is a critically endangered Plecoptera species. Considered extinct in the UK, *I. nubecula* was recently rediscovered (in one location of the River Dee, Wales), after 22 years of absence. In a similar way to many other species of Perlodidae, *I. nubecula* could be utilised as a bio-indicator, for assessing water quality and health status of a given freshwater system. However, conventional monitoring of invertebrates via kick-sampling, is invasive and expensive (time consuming). Further, such methods require a high level of taxonomic expertise. Here, we compared the traditional kick-sampling method with the use of eDNA detection using qPCR and ddPCR-analyses. In spring 2018, we sampled eDNA from twelve locations on the River Dee. *I. nubecula* was detected using kick-sampling in five of these locations, three locations using both eDNA detection and kick-sampling and one location using eDNA detection alone – resulting in a total of six known and distinct populations of this critically endangered species. Interestingly, despite the eDNA assay being validated *in vitro* and *in silico*, and results indicating high sensitivity, qPCR analysis of the eDNA samples proved to be ineffective. In contrast, ddPCR analyses resulted in a clear detection of *I. nubecula* at four locations suggesting that inhibition most likely explains the large discrepancy between the obtained qPCR and ddPCR results. It is therefore important to explore inhibition effects on any new eDNA assay. We also highlight that ddPCR may well be the best option for the detection of aquatic organisms which are either rare or likely to shed low levels of eDNA into their environment.

## Introduction

Monitoring biodiversity in freshwater systems is a cornerstone of the evaluation of the European Habitats Directive, the European Water Framework Directive and the general evaluation of ecosystem health and status^1–3^. The assessment of freshwater biodiversity relies on biological monitoring methods, in which the use of biodiversity indicators is an essential component of its evaluation. Various aquatic macroinvertebrates, such as mayflies, stoneflies and caddisflies (Ephemeroptera, Plecoptera and Trichoptera) are commonly used as bio-indicator organisms for water quality and ecosystem assessments^4–6^. This is down to how they react to anthropogenic change such as pollution, climate change, fracking, mining, and the construction of hydroelectric stations for example^5,7–9^.

Traditional monitoring of macroinvertebrates via kick-sampling and/or capture-recapture methods, is, however, costly (i.e. time consuming), labour intensive and, above all, known to be limited in effective detection of populations below a certain threshold^5,10^. Further, such methods are invasive ecologically speaking i.e. they increase the risk of injury to the target (and non-target) organism or organisms. The morphological identification of these bio-indicators is also often challenging, especially at immature life stages^5,11–13^, therefore a high level of taxonomic expertise is often required in order to avoid any possible misidentification and therefore misrepresentation^14,15^.

The use of molecular approaches for bio-monitoring, such as the detection of environmental DNA (eDNA), may overcome a number of these issues^16^. Moreover, the use of eDNA increases efficiency, reliability and allows for a more rapid species identification and ultimately detection^5^. Whilst minimising any associated impacts on the species and the environment. All aquatic organisms shed DNA traces into their environment^17^, and it is now possible to detect a specific species (barcoding) or assess an entire community (metabarcoding) by sampling an aquatic system and amplifying the existing DNA traces using Polymerase Chain Reaction (PCR)^17^. eDNA based methods have now been designed and proven successful for monitoring invasive^18–22^, endangered^23,24^ and/or economically important species from a wide range of taxa^25–27^. However, few studies have used eDNA for monitoring rare or indicator macroinvertebrate species^28–30^.

A typical example of a bioindicator Plecoptera is the Scarce Yellow Sally stonefly, *Isogenus nubecula* (Perlodidae, Plecoptera) (Newman 1833). This critically endangered species has been reported as extinct or undetected in much of its historical home range^31,32^. *I. nubecula* is listed as a UK Biodiversity Action Plan priority species and a species of principal importance in Wales (www.nhm.ac.uk/our-science/data/uk-species/species/isogenus_nubecula) for example. Indeed, across the UK, *I. nubecula* was thought to be extinct until a population was recently discovered (in the River Dee, North Wales) after a 22 year period of absence^32^. The aim of this study was to design a novel single species eDNA based assay for the detection of *I. nubecula* and compare the efficiency of quantitative Polymerase Chain Reaction (qPCR) and droplet digital Polymerase Chain Reaction (ddPCR) versus traditional kick-sampling.

## Results

### Specificity and validation of eDNA assay using PCR, qPCR and ddPCR

**Figure 1:**
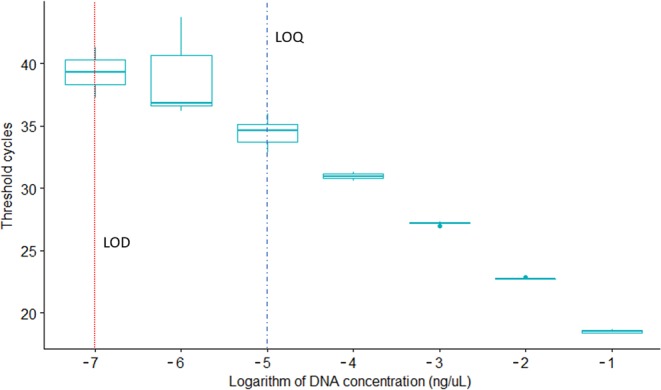
Standard curve assessing the Limit of Detection (LOD) and Limit of Quantification (LOQ) for the qPCR assays detecting the DNA traces of I. nubecula. Both limits were calculated from a 1:10 serial dilution with 10 replicates per concentration. The LOD was 6.82 10^−6^ ng DNA µL^−1^ at 39.29 ± 2.00 Ct (i.e. Cycle threshold) and the LOQ was 6.82 10^−4^ ng DNA µL^−1^ at 34,48 ± 0,95 Ct (Slope = −3.86, Y inter = 19.52, R2 = 0.97, Eff% = 81.63).

The eDNA assay (primers and probe) designed in this study were species-specific *in-silico* and *in-vitro* with both conventional PCR and qPCR. The negative controls or samples with DNA from non-target species did not amplify with either method. For qPCR, we analysed the standard curve and compiled the limit of detection (LOD) and limit of quantification (LOQ) as per the MIQE guidelines^33,34^. The LOD was 6.82 × 10^−6^ ng DNA µL^−1^ at 39.29 ± 2.00 Ct (i.e. Cycle threshold) and the LOQ was 6.82 × 10^−4^ ng DNA µL^−1^ at 34.48 ± 0.95 Ct (Slope = −3.86, Y inter = 19.52, R^2^ = 0.97, Eff% = 81.63) (Fig. 1). With ddPCR, five of the replicates from the dilution, equating to 0.08 pg of DNA yielded a positive detection for *I. nubecula* (mean 0.05 copy per µL^−1^). Interestingly, only one replicate from the next dilution series (0.016 pg) yielded a positive detection (0.08 copy per µL^−1^). All further dilutions and the negative controls were negative. However, as shown in other studies^35^, the lower LOD readings (with a 95% confidence level) can sometimes overlap with apparent artefacts seen in the negative controls. For this reason, we considered 0.08 pg of DNA to be the lowest amount able to be detected using ddPCR and only considered samples >0.5 copy per µL^−1^ (as in^35^) to meet the threshold for a positive detection.

### Kick sampling assessment

Populations of *I. nubecula* were identified at five different locations along the River Dee, and apparently absent at a further five (Fig. 1, Table 1). Abundance ranged from just one individual at two of the sites (W7 and W8), up to a highest density of 30 individuals at W3. Two of the sites (surveyed using the eDNA assay) were unable to be assessed via kick sampling due to dangerous access and weather conditions at the time of sampling (Table 1).

**Table 1:** Table depicting the kick-sampling results for *I. nubecula* (i.e. how many specimens found at each site), the eDNA results using ddPCR analysis (i.e. if one natural replicate was positive to *I. nubecula* DNA), the amount of time spent performing kick-sampling and eDNA sampling, the amount of water filtrated for all natural replicate at each site, the sampling date, pH, dissolved oxygen and GPS coordinate. The site inaccessible for conducting a kick-sampling were marked “ns”.

Sample ID	*I. nubecula*	eDNA (ddPCR)	Time (s)	Volume (ml)	Date	pH	O_2_	Latitude	Longitude
W1	0	No	60	350	31/03/2018	7.48	12.5	52.952759	−3.0232733
W2	3	Yes	60	200	01/04/2018	7.53	11.9	53.024980	−2.8760059
W3	30	No	60	700	09/03/2018	6.69	11.9	53.010679	−2.8998019
W4	16	Yes	120	1000	09/03/2018	6.52	11.8	53.003120	−2.9138314
W5	ns	Yes	45	750	15/03/2018	7.83	11.4	53.095257	−2.8967275
W6	ns	No	60	300	01/04/2018	7.82	12.5	53.011702	−2.8686273
W7	1	Yes	90	750	14/03/2018	7.67	11.6	52.978139	−2.9627502
W8	1	No	90	750	14/03/2018	7.8	10.7	52.964635	−2.9628967
W9	0	No	90	750	11/03/2018	6.75	11.8	52.945402	−3.0194684
W10	0	No	60	300	31/03/2018	7.74	13	52.970460	−3.0879607
W11	0	No	45	500	11/03/2018	6.63	11.6	53.100487	−2.9239146
W12	0	No	90	750	15/03/2018	7.69	10.9	52.967603	−3.0619060

### Comparison of qPCR versus ddPCR analyses

**Figure 2:**
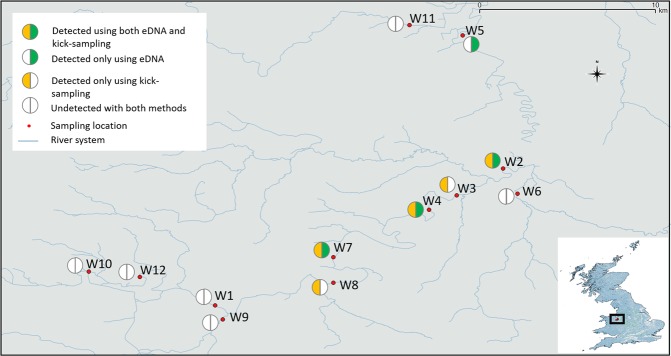
Map showing the 12 locations of the River Dee sampled with both kick-sampling and eDNA survey for monitoring I. nubecula in Wales, United Kingdom. Red dots are showing the sampled locations, half green circle the locations positive with eDNA detection using ddPCR, the half orange circle the locations were *I. nubecula* was found using kick-sampling. Locations W5 and W6 were not surveyed using kick-sampling method.

Despite the success of the assay (*in-silico* and *in-vitro*), we were unable to amplify DNA via qPCR in any of the eDNA samples (Table 2). This was even true of the ‘positive control eDNA sample’ which consisted of 11 *I. nubecula* individuals housed in a 1 litre mesocosm for a period of one hour before filtering (see methods). During each run, the positive dilution range indicated the assay ran without any issue (Slope = −3.65/−4.05, Y inter = 19.22/26.46, R^2^ = 0.98/0.99, Eff% = 76.46/88.03). In contrast, the ddPCR analysis revealed a positive detection of *I*. *nubecula* at four sampling locations (Fig. 2, Table 2). Interestingly, only two of the sites (W4 and W5) were positive with both undiluted DNA and diluted template (1:2). W2 was positive only when we utilised undiluted eDNA, and W7 only when we diluted 1 in 2 (Table 2). Concentration of eDNA was relatively low and ranged from 0.6 to 0.14 copies per µL^−1^ across all samples. The ‘positive eDNA’ sample generated a much higher DNA concentration of 5.4 copies per µL^−1^ (undiluted) and 8.2 copies per µL^−1^ (diluted).

**Table 2:** Table depicting the eDNA detection results using qPCR and ddPCR techniques on diluted and undiluted (1:2) natural replicates (NR) sampled at each field location.

Sample ID	qPCR			ddPCR					
	undiluted			undiluted			diluted		
	NR A	NR B	NR C	NR A	NR B	NR C	NR A	NR B	NR C
W1	—	—	—	—	—	—	—	—	—
W2	—	—	—	—	0.8	0.7	—	—	—
W3	—	—	—	—	—	—	—	—	—
W4	—	—	—	—	—	0.7	0.7	—	0.14
W5	—	—	—	0.7	0.7	0.14	—	—	0.6
W6	—	—	—	—	—	—	—	—	—
W7	—	—	—	—	—	—	—	0.7	—
W8	—	—	—	—	—	—	—	—	—
W9	—	—	—	—	—	—	—	—	—
W10	—	—	—	—	—	—	—	—	—
W11	—	—	—	—	—	—	—	—	—
W12	—	—	—	—	—	—	—	—	—
‘positive control’	—			5.4			8.2		

‘—’ depict the absence of eDNA detection using qPCR and/or ddPCR. Quantification values of ddPCR results are displayed in copy per µL^−1^. Natural replicates were analysed using six technical replicates with qPCR and without replicates using ddPCR. All samples revealed a negative result for *I. nubecula* eDNA using qPCR. DNA from the targeted specie was amplified in samples from four field locations and in the ‘positive control’.

The site occupancy modelling approach did not reveal any significant effect of the environmental variables on the presence of eDNA or on the probability of detection (Tables 3 and 4). Probabilities of *I*. *nubecula* occurrence were relatively low and ranged from 0.45 to 0.53 (Table 4). Probabilities of eDNA detection at each sampling site ranged from 0.59 at site W5 (where all ‘natural replicates’ where found to be positive using ddPCR) to 0.27 at site W10, a site with high turbidity where no stonefly were found.

**Table 3:** Table depicting the Bayesian estimates for effects of covariates on the probability of occurrence at a site (*ψ*).

Bayesian estimates of model parameters				
	Mean	50%	2.5%	97.5%
β Intercept	0.135	0.086	−1.107	1.610
β Accessibility	−0.232	−0.189	−1.678	1.113
α Intercept	0.970	0.933	−0.265	2.506
α Volume	0.151	0.166	−1.026	1.178
α pH	0.156	0.134	−1.118	1.671
δ Intercept	−0.136	−0.136	−0.847	0.619
δ Volume	0.275	0.292	−0.486	1.054
δ O_2_	−0.037	−0.087	−2.037	2.102
δ Time	−0.149	−0.153	−0.845	0.575
**Monte Carlo SE of Bayesian estimates**				
	**Mean**	**50%**	**2.5%**	**97.5%**
β Intercept	0.0345	0.0418	0.0420	0.0316
β Accessibility	0.0305	0.0372	0.0305	0.0474
α Intercept	0.0391	0.0434	0.0516	0.0332
α Volume	0.0220	0.0258	0.0439	0.0302
α pH	0.0306	0.0342	0.0407	0.0462
δ Intercept	0.0166	0.0189	0.0204	0.0255
δ Volume	0.0156	0.0182	0.0225	0.0178
δ O_2_	0.0667	0.0704	0.0840	0.0819
δ Time	0.0188	0.0199	0.0207	0.0249

(*α*) and (*δ*) parameters are covariates for the conditional probability of eDNA presence in a sample (θ) and for its detection (*p*). (*β*) parameters are covariates of the estimated occupancy (*ψ*). Means represent estimated parameter values and last two columns represent the boundaries of the 95% credible intervals.

**Table 4:** Table depicting the Bayesian estimates for the probabilities of occurrence (*ψ*), the conditional probabilities of eDNA presence in a sample (*θ*) and eDNA detection (*p*) of *I. nubecula* at each sampling site of the River Dee and its tributaries.

Site	*ψ*	*θ*	*p*
W1	0.45	0.79	0.33
W2	0.45	0.76	0.31
W3	0.45	0.77	0.52
W4	0.45	0.75	0.48
W5	0.53	0.87	0.59
W6	0.53	0.81	0.32
W7	0.45	0.86	0.46
W8	0.45	0.87	0.52
W9	0.45	0.77	0.47
W10	0.45	0.80	0.27
W11	0.45	0.75	0.49
W12	0.45	0.86	0.50

## Discussion

In our study, we compared the use of kick-sampling and eDNA detection for monitoring a critically endangered bioindicator macroinvertebrate. While our eDNA detection approach using qPCR showed high sensitivity (Fig. 1), with no false positive results during the validation process and assessment of the MIQE guidelines (Appendix 2),we were, however, not able to amplify DNA traces of *I. nubecula* in any of the eDNA samples. This is surprising as one should expect positive detection at least in the five locations where we found the species via kick-sampling, and especially in the’positive eDNA’ sample. These observations thus clearly pose doubts on the concept of eDNA using the qPCR methodology. Potential explanations for these false negative observations might be (i) an incorrect sampling protocol, (ii) the presence of PCR inhibitors in the DNA extracts, or (iii) a very limited shedding rate of the targeted species^36^. As previously shown, the sampling design of any eDNA based study can affect the reliability of detection^34^. In this case, we accounted for this by taking three natural replicates at each site and incorporating six technical PCR replicates per sample. Therefore, we believed our sampling protocol to be sufficient.

This left inhibition of the qPCR assay as the most likely reason for the false negative detections, as has been shown in a number of other studies^37,38^. One can assess for inhibition via the use of internal positive controls, such as spiked synthetic DNA or DNA from organisms different than the targeted species^36^. Limited detection or complete failure of such internal controls may then clearly show the occurrence of inhibition factors. If there is inhibition, two methods can be utilised to overcome this issue. The first method is to dilute the DNA extracted from the field sample^36^, whilst the second is the use of an inhibitor removal kit^36,39^. However, both methods have been shown to reduce the yield of target DNA in the extracted sample^36^. In our study, qPCR showed no results from the eDNA samples and so we hypothesised that inhibition may be an important driver for the false negative observations in this assay. We did not use an inhibitor removal kit, as we wanted to  avoid reducing the amount of DNA extracted from the field samples. Instead, we opted to run the samples on a ddPCR with two different dilutions. The use of ddPCR worked and four sites revealed a positive signal, three of which mapped with the results from our kick-sampling survey. However, the influence of diluting the eDNA extracts also became apparent when analysing the results. eDNA was shown to be positive for only two of the sites (W4 and W5) regardless of dilution, one (W2) was positive only with undiluted eDNA template and another (W7) only when the extract was diluted 1:2 (Table 2). This result indicates care should be taken with regard to dilution of extract in future eDNA-based studies (both for qPCR and ddPCR) and where possible multiple dilutions (starting from zero) should be run to give greater confidence in the results.

It is not surprising that ddPCR outperformed qPCR in this study and similar results have been shown before^40–43^. This is simply because ddPCR partitions any given sample into thousands of droplets, performing independent end-point PCR amplification on each droplet, thereby enabling the detection and quantification of very low amounts of DNA^41^. After amplification, the fluorescence of each droplet is measured allowing quantification of the targeted DNA (Bio-Rad’s QuantaSoft software version 1.7.4.0917). This is in contrast to qPCR, which relies on the detection of PCR amplification, rather than amplification efficiency. Interestingly, the analysis of the ‘positive eDNA sample’ (11 *I. nubecula* in a 1 litre mesocosm for a period of one hour before filtering) showed an increase from 5.4 copies per µL^−1^ (undiluted) to 8.2 copies per µL^−1^ (diluted). This indicates that inhibition is still affecting the ddPCR (although not strong enough to block amplification in this instance). Future research should therefore explore the role of inhibition in eDNA based methods as a matter of urgency to ensure confidence in these tools remains high.

The very low *I. nubecula* eDNA concentrations in the samples (0.6 to 0.14 copies per µL^−1^) and the still relatively low concentration of eDNA in the ‘positive eDNA’ sample (8.2 copies per µL^−1^) indicates that this species may have very low shedding rates. However, as this is the first study to utilise ddPCR for detecting low populations of an endangered invertebrate in a fast-flowing river, we are unable to compare our results with other studies to date. Besides the fact that invertebrates are generally found to shed only limited amounts of eDNA in the water, potential other explanatory variables of these low levels of eDNA for this species could be the high flow rate of the river and low temperature during sampling. Sample were collected at the end of winter/beginning of spring, when environmental conditions such as high flow rates or flood events could have decreased and diluted the quantity of DNA traces. However, this was unavoidable for this species as *I. nubecula* emerges from March onwards^31,44^ and so sampling time could not be altered.

**Figure 3:**
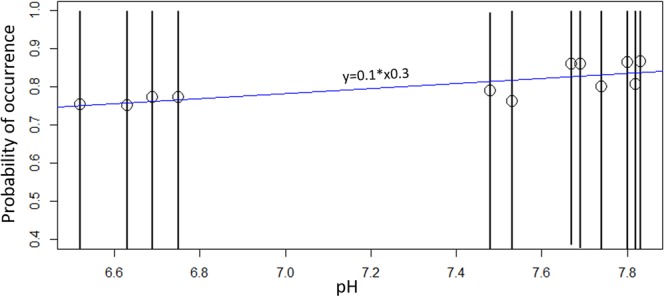
Estimated probability of occurrence of *I. nubecula* eDNA with the pH of each sampling sites. Dots are representing each sampling locations, the black lines are representing the estimates of posterior medians with 95% credible intervals and the blue line the regression analysis.

Finally, when sampling for any eDNA study, it is useful to have an understanding of the ecology of the species under study, such as the species habits and preferred habitat in which it occurs. However, again, as *I. nubecula* was only recently rediscovered in the UK, there is very little information on this species^32^. Our site occupancy modelling approach was also unable to identify any specific variable that would have a significant effect on the probability of detection of this species using our eDNA assay (Fig. 3, Tables 3 and 4). This was not surprising however, as all the individual sites were on the same river system. Indeed, occupancy modelling is known to have certain limitations, mainly driven by the number of locations sampled and restricted range of environmental values collected^43^. In addition, the rarity of our target organism in this study and its likely high stochasticity (with regard to population distribution) would influence the models’ outputs and ultimate usefulness. Thereby exploring the effects of the underlying environmental drivers on the distribution of *I. nubecula* remains difficult at the current time. Further work will therefore be necessary in order to increase our understanding of the ecology of *I. nubecula* if we want to optimize the sampling protocol and conservation plans for this species. However, the application of site occupancy modelling can become beneficial when prior survey data is combined with a more intensive survey effort. In such cases, a more informed post experimental understanding will be obtained^45,46^.

In conclusion, even if the highest standards of validation are undertaken in the design and implementation of an eDNA based PCR or qPCR assay^28–30^, false negative results can and do appear, driven by inhibition factors^36^, low shedding rates from the target species^18,47^ and/or low population sizes^20^. In this case we are dealing with an extreme scenario, in which none of our eDNA samples showed any amplification via qPCR despite the fact that populations of *I. nubecula* were known to be present. However, we were able to get positive detection (using ddPCR) at five independent sites, three of which mapped against a physical detection of the species using kick sampling. Less than ten studies have (at the time of writing) utilised this technology for eDNA assays^35,40–43,48–50^, but this is likely to increase significantly due to the apparent benefits observed in this study for example. We end by highlighting that negative results, derived from assays reliant solely on qPCR should be viewed with caution, for the reasons given above.

## Methods

### Primers and probe design

A species-specific set of primers and probe, targeting the COI gene (Cytochrome C Oxidase subunit 1 mitochondrial gene) of *I. nubecula* was designed using the Geneious Pro R10 Software (https://www.geneious.com ^51^. This assay amplifies a 124 bp fragment using the forward primer (5′–CCAGAAGCCTTGTAGAAAAC–3′), the reverse primer (5′–ACCCCGGCTAGATGAAGAGA–3′) and a probe (6-FAM–CCCCACTCTCTGCTGGAATT–BHQ-1). Specificity of the assay was assessed *in-silico* by comparing against sequences from 21 genetically similar invertebrate species, previously submitted to the NCBI (National Centre for Biotechnology Information; https://www.ncbi.nlm.nih.gov/) see Appendix 1 for full list. The specificity of the assay was tested *in-vitro* using PCR and qPCR, with DNA extracted from the nine invertebrate species (closely related or likely to be present in the same ecosystem). These included; *I. nubecula*, *Leuctra hippopus* (Kempny, 1899), *Perlodes mortoni* (Klapálek, 1906), *Nemoura lacustris* (Pictet, 1865), *Leuctra geniculata* (Stephens, 1836), *Nemoura erratica* (Claassen, 1936), *Taeniopteryx nebulosa* (Linnaeus, 1758), *Diura bicaudata* (Linnaeus, 1758) and *L. fusca* (Linnaeus, 1758).

### eDNA samples

12 locations from the River Dee, were sampled for eDNA between 9^th^ March 2018 and 1^st^ of April 2018 (Fig. 1 and Table 1). These locations were chosen following previous knowledge of historical observations in 1981 and 1982^32^. At each location, three independent (i.e. A, B and C) 1 L water samples (referred to here after as natural replicates) were collected using a 40 mL sterile polypropylene ladle and placed into a sterile plastic bag (Whirl-Pak® 1242 ml Stand-Up Bag Merck®, Darmstadt, Germany)^34^. Sub-samples were regularly collected from surface water downstream to upstream (to avoid disturbing sediments), across the width or the bank of the river, depending on the access and weather conditions following the method outlined in^52^. Each independent 1 L water sample was then filtered with a sterile 50 mL syringe (sterile Luer-Lock™ BD Plastipak™, Ireland) through a sterile 0.45 μm Sterivex™ HV filter (Sterivex™ filter unit, HV with luer-lock outlet, Merck®, Millipore®, Germany). Sterivex filters were immediately placed in a freezer bag and stored at −80 °C until further analysis. At each location, new sterile equipment and disposable nitrile gloves were used during the sampling process to avoid contamination. A ‘positive’ eDNA sample was collected by creating an isolated mesocosm onsite, which consisted of river water from site W4 and 11 specimens of *I. nubecula* stored for 1 hour. Two negative control samples were additionally filtered in the field with sterile ddH_2_O in parallel with the natural samples, to control for potential cross-contamination during the workflow.

### DNA extraction

DNA extraction from both the eDNA samples and the tissue samples (utilised for validating the assay) was done using the Qiagen DNeasy® Blood and Tissue Kit. We followed the manufacturer’s instructions for performing DNA extraction from tissue samples. Sterivex filters were extracted following the methods outlined in^53^). All laboratory equipment was disinfected and decontaminated using UV-treatment prior to conducting any laboratory work. Laboratory equipment and surfaces were regularly disinfected using 10% bleach and absolute ethanol before conducting analyses.

### PCR

PCR amplifications were performed on a Gen Amp PCR System 9700 (Applied Biosystem) using the primers described above. PCR reactions were performed in a 25 µL reaction, with 12.5 µL of PCRBIO Ultra Mix Red (PCRBIOSYSTEMS), 1 µL of each primer (10 µM), 9.5 µL of ddH_2_O and 1 µL of template DNA. Optimal PCR conditions were performed under thermal cycling 50 °C for 2 min and 95 °C for 10 min, followed by 35 cycles of 95 °C for 15 s and 60 °C for 1 min. For each PCR (with DNA from tissue samples), at least one positive and one negative control were included.

### qPCR

qPCR amplifications were performed on an ABI StepOnePlus™ Real-Time PCR (Applied Biosystems) in final volumes of 25 µL, using 12.5 µL of PrecisionPlus qPCR Master Mix with ROX (Primer Design, UK), 1 µL of each primer (10 µM), 1 µL of probe (2.5 µM), 6.5 µL of ddH_2_O and 3 µL of extracted DNA. qPCR conditions were as follow: 50 °C for 2 min and 95 °C for 10 min, followed by 45 cycles of 95 °C for 15 s and 60 °C for 1 min. For each qPCR with DNA from tissue samples, at least two positive and two negative controls were included. A standard curve was established by analysing a 1:10 dilution series of DNA extracted from *I. nubecula* (68.2 ng/ µL, Nanodrop 2000 Spectrophotometer, ThermoFisher Scientific) following the MIQE Guidelines^33^ (Appendix 2).

### ddPCR

Digital droplet PCR was conducted using the Bio-Rad QX200 ddPCR system in a 20-μl total volume. Each reaction contained 10 μL Bio-Rad ddPCR supermix for probes (no dUTP), 750 nM of each primer, 375 nM probe, 3 µL DEPC water, and 4 µl template DNA. Twenty microlitres of the PCR mix was pipetted into the sample chambers of a Droplet Generator DG8 Cartridge (Bio-Rad, cat no. 1864008), and 70 μL of the Droplet Generation Oil for Probes (Bio-Rad, Cat No. 186-4005) was added to the appropriate wells. The cartridges were covered with DG8 Gaskets (Bio-Rad, cat no. 1863009) and placed in a QX200 Droplet Generator (Bio-Rad) to generate the droplets. After droplet generation, the droplets (40 μL) were carefully transferred to a ddPCR 96-well plate (Bio-Rad, Cat No. 12001925). The PCR plates were sealed with pierceable foil (Bio-Rad, Cat No. 181-4040). PCRs were performed using a C1000 Touch^TM^ Thermal Cycler with a 96-well Deep Reaction Module (Bio-Rad). PCR conditions were 10 min at 95 °C, followed by 40 cycles of denaturation for 30 s at 94 °C and extension at 60 °C for 1 min, with ramp rate of 2 °C s-1, followed by 10 min at 98 °C and a hold at 12 °C. Droplets were then read on a QX200 droplet reader (Bio-Rad). All droplets were checked for fluorescence and the Bio-Rad’s QuantaSoft software version 1.7.4.0917 was used to quantify the number of *I. nubecula* copies per µL. Thresholds for positive signals were determined according to QuantaSoft software instructions. All droplets beyond the fluorescence threshold (3500) were counted as positive events, and those below it as negative events. All eDNA samples were analysed in duplicate (one replicate undiluted and one replicate diluted 1:2). One positive control (i.e. DNA extracted from *I. nubecula* at a concentration of 1 ng/µL diluted 1:100 (Nanodrop 2000 Spectrophotometer, ThermoFisher Scientific)), one No Template Control (i.e., IDTE pH 5.0) and the two negative field controls were additionally included. The LOD using the ddPCR was assessed following the method outlined in^35^. We conducted a serial dilution of a DNA extracted from *I. nubecula*. The starting point was an initial 1: 100 dilution of extracted genomic DNA from *I. nubecula* at 1 ng/µL, followed by a serial 1:5 dilution. The serial dilution included ten replicate of each dilution.

### Estimation of the LOD and LOQ

To attain estimates of the LOD and LOQ for the primers and probe for both qPCR and ddPCR, we set-up a dilution range from 10^−1^ to 10^−9^ with 10 technical replicates used for each of the dilution steps. Following^34^, the LOD was defined as the last standard dilution when the targeted DNA was detected and quantified in at least one replicate with a threshold cycle under 45. The LOQ was defined as the last standard dilution in which the targeted DNA was detected and quantified in at least 90% of positive samples^34,52^. All eDNA samples were then analysed with six technical replicates^34,52^ on a qPCR plate, with six negative controls and a positive control dilution series from 10^−1^ to 10^−6^ in duplicate.

### Kick-sampling

Kick-sampling was performed using the standard of the Freshwater Biological Association (UK), i.e. using a kick-sampling net with a 1 mm mesh (see detailed protocol: https://www.fba.org.uk/practical-guidance-sampling-and-collecting). Sampling duration was recorded at each site but varied dependant on access, depth, flow rate, and/or weather conditions (Table 1). Perlodidae specimens found during kick-sampling were either preserved in 99% ethanol or kept alive as a part or a separate rearing experiment. Specimens were identified in the laboratory by two independent taxonomy experts (John Davy-Bowker & Michael Hammett) using a low-power binocular microscope with cold light source and using an identification key^44,54^.

### Statistical analysis

A site occupancy modelling approach^45,55,56^ was utilised to assess the effect of environmental covariates on the presence of eDNA of *I. nubecula* and to estimate the detection probability of the new assay. This hierarchical modelling framework has the advantage of accounting for the risk of false negative results when estimating the probability of detection. This analysis was run with the ddPCR data (Appendix 3). Covariates tested included: (i) turbidity (likely to inhibit the PCR reaction, with the volume of filtered water being used as a proxy), (ii) pH, (iii) dissolved oxygen concentration, (iv) amount of time spent at each location (for both eDNA sampling and kick-sampling)used as a proxy for field conditions and (v) site accessibility as a binary indicator (possible to perform kick-sampling/absence of kick-sampling survey) (Appendix 4). Analyses were performed using the ‘eDNAoccupancy’ package^43,57^ in the R statistical programming environment (R Core Team, 2018). Model selection and interpretation followed procedures given in^43,57^. We fitted our model using the ‘occModel’ function from the described package. MCMC chains ran for 11,000 iterations, with 10,000 retained for obtaining parameter estimates and credible intervals.

## Supplementary information

Annexes
